# Maintenance tegafur-plus-uracil after adjuvant concurrent chemoradiotherapy may improve outcome for resected oral cavity squamous cell carcinoma with extranodal extension

**DOI:** 10.3389/fonc.2022.866890

**Published:** 2022-09-28

**Authors:** Pei-Wei Huang, Chien-Yu Lin, Li-Yu Lee, Chia-Hsun Hsieh, Cheng-Lung Hsu, Chi-Ting Liau, Kang-Hsing Fan, Shiang-Fu Huang, Chun-Ta Liao, Tung-Chieh Chang, Hung-Ming Wang

**Affiliations:** ^1^ Division of Medical Oncology, Department of Internal Medicine, Chang Gung Memorial Hospital at Linkou, Taoyuan, Taiwan; ^2^ Departments of Radiation Oncology, Chang Gung Memorial Hospital at Linkou, Taoyuan, Taiwan; ^3^ College of Medicine, Chang Gung University, Taoyuan, Taiwan; ^4^ Department of Pathology, Chang Gung Memorial Hospital at Linkou, Taoyuan, Taiwan; ^5^ Section of Head and Neck Surgery, Department of Otorhinolaryngology, Chang Gung Memorial Hospital at Linkou, Taoyuan, Taiwan

**Keywords:** tegafur plus uracil, maintenance therapy, head and neck cancer, extranodal extension, oligometastasis

## Abstract

**Objectives:**

To evaluate whether tegafur-uracil maintenance (UFTm) following postoperation adjuvant cisplatin-based concurrent chemoradiotherapy (CCRT) may reduce distant metastasis in patients with resected oral cavity squamous cell carcinoma (OSCC) with pathologic extranodal extension (pENE+).

**Methods:**

A retrospective comparison was conducted between two cohorts of patients with resected pENE+ OSCC who completed adjuvant CCRT between March 2015 and December 2017, including one cohort of a phase II trial using UFTm and a trial-eligible but off-protocol cohort without using UFTm (non-UFTm) after their adjuvant CCRT. The UFTm trial enrolled patients without relapse within 2 months after the end of adjuvant CCRT and administered UFT 400 mg/day for 1 year. Kaplan–Meier methods estimated the actuarial rate of distant metastasis-free (DMF), locoregional control (LRC), event-free survival (EFS), and overall survival (OS).

**Results:**

A total of 103 patients were included in this study, 64 patients in UFTm and 39 patients in non-UFTm. Severe adverse events in UFTm included grade 3 anemia (n = 1, 1.6%) and grade 3 mucositis (n = 1, 1.6%). A total of 40 (62.5%) patients completed the full course of UFTm, while the remaining terminated UFTm earlier due to disease relapse (n = 14, 21.8%), poor compliance (n = 9, 14.1%), and adverse event (n = 1, 1.6%). The median (range) follow-up time of surviving patients was 43 (22–65) months. The outcomes compared between UFTm and non-UFTm were OS (hazard ratio [HR] 0.31 [95% CI: 0.17–0.57], p < 0·001), EFS (0.45 [0.25–0.82], 0.009), LRC (0.45 [0.19–1.05], 0.067), and DMF (0.47 [0.24–0.95], 0.035). Multivariable analysis, adjusted for UFTm, Charlson comorbidity index score 1–3, site of tongue, and number of ENE+ LN ≧4, confirmed better OS (0.29 [0.16–0.54], <0.001) and EFS (0.47 [0.26–0.85], 0.012) in favor of UFTm over non-UFTm. The 2-year DM rate was 25.8% in UFTm and 44.2% in non-UFTm. For relapsed patients in UFTm vs. non-UFTm, the rate of metastasectomy for oligometastasis was 53% vs. 6%, and the OS was 21.0 (95% CI: 17.8–24.1) months vs. 11.0 (9.1–12.8) months (p < 0.001), respectively.

**Conclusions:**

UFTm may improve the dismal outcomes of the resected pENE+ OSCC. Further investigations are needed to confirm our observations.

## Introduction

According to the GLOBOCAN estimates, head and neck cancer was the seventh most common cancer worldwide, with 890,000 new cases and 450,000 deaths reported in 2018 (1). Taiwan is an endemic betel quid-chewing area. Oral cancer accounts for the third highest cancer incidence (42.59/10^6^) and the fourth rate of death by cancer (17.15/10^6^) in Taiwanese men, with two-thirds of oral cancer arising within the oral cavity (2). Surgical treatment remains the standard upfront therapy for oral cavity squamous cell carcinoma (OSCC). Adjuvant cisplatin-based concurrent chemoradiotherapy (CCRT) is the current standard practice for resected head and neck squamous cell carcinoma (HNSCC) with pathologically documented extranodal extension (pENE) and/or positive margin (3–5).

pENE reflects aggressive tumor behavior (6) and has been incorporated to refine the risk stratification of TNM N classifications (7). The presence of pENE increases the possibility of tumor cells entering the blood stream, which, in turn, increases the risk of distant metastasis (DM) (8). Our previous study, focusing on 345 patients with resected OSCC and positive lymph nodes (LNs), revealed a distant failure rate of 39% in pENE-positive patients (pENE+) vs. 12% in pENE-negative patients (hazard ratio [HR] 3.50 (95% confidence interval [CI], 2.08–5.88), p < 0.001) (9). Current adjuvant cisplatin-based CCRT for high-risk resected HNSCC targeting pENE+ and/or positive resection margin improved locoregional control (LRC), progression-free survival (PFS), and overall survival (OS) but did not reduce the 25%–30% distant failure rate (3–5).

Efforts to reduce distant failure in the adjuvant setting of resected HNSCC are still lacking. The serendipitous discovery of reduced distant failure in resected HNSCC had been demonstrated by pre-radiotherapy durable cisplatin plus 5-fluorouracil (5-FU)-combined chemotherapy in the Intergroup 0034 trial (10), docetaxel plus cetuximab during adjuvant CCRT in the RTOG 0234 trial (11), and adjuvant tegafur-uracil (UFT) alone (without radiotherapy) in the Head and Neck UFT Study Group (12). The reduced distant failure rates found in the trials were 23% vs. 15% (p = 0.03) in the intergroup trial (10), 25% vs. 13% (p = 0.03) in the RTOG 0234 trial (11), and 14.6% vs. 7.9% (p = 0.03) in the UFT trial (12). However, having seen no improvement in LRC, disease-specific survival (DSS), or OS in these trials, impedes further investigations.

UFT contains tegafur and uracil in a molar ratio of 1:4. Tegafur is metabolized *in vivo* to 5-FU, and uracil helps maintain the intracellular levels of 5-FU by inhibiting its degradation (13). It was reportedly effective in treating HNSCC in a recurrent/metastatic setting (14), and in CCRT (15) and neoadjuvant settings (16). In addition to the chemotherapeutic effect, preliminary data from studies indicate a possible anti-angiogenic activity of UFT (13, 17). These characteristics have led to UFT being recommended and clinically used as metronomic therapy that could be used as maintenance therapy after adjuvant therapy for high-risk resected HNSCC. The minimal adverse events associated with UFT further support its long-term use.

With the above rationale for using UFT in reducing distant failure, we conducted a retrospective comparison between two cohorts of patients with or without UFTm after their standard adjuvant CCRT for resected pENE+ OSCC treated during March 2015 and December 2017.

## Materials and methods

In this retrospective study, data from patients with resected pENE+ OSCC with or without maintenance UFT (UFTm) after their standard adjuvant CCRT between March 2015 and December 2017 were collected and analyzed. The UFTm cohort was originally enrolled in the phase II UFTm trial (NCT03121313) for patients with resected pENE+ OSCC after adjuvant CCRT. The cohort without UFTm (non-UFTm) consisted of patients with resected pENE+ OSCC fulfilling the UFTm trial criteria who were treated during the same period but did not participate the UFTm trial after their adjuvant CCRT.

The inclusion criteria for participants in the phase II UFTm trial were age 20–70 years, Eastern Cooperative Oncology Group (ECOG) performance status score 0 or 1, adequate hematopoietic and organ function, histologically confirmed OSCC, post-definitive surgical treatment status with pENE, adjuvant cisplatin-based CCRT commenced within 6–8 weeks after the definitive surgical treatment, completed adjuvant CCRT course with radiotherapeutic dose ≥6,000 cGy, no common terminology criteria for adverse events (CTCAE) 4.03 grading ≥2 acute adverse events following previous definitive treatment, and no DM at enrollment. Adequate hematopoietic function was defined as white blood cell ≥3,000/mm^3^, absolute neutrophil count ≥1,500/mm^3^, and platelet count ≥100,000/mm^3^. Adequate organ function was defined as serum bilirubin level <1.5 × upper limit of normal (ULN), serum glutamic oxaloacetic transaminase and serum glutamic pyruvic transaminase <2.5 × ULN, and serum creatinine level <1.5 × ULN. The pENE was defined in accordance with the American Joint Committee of Cancer (AJCC) 8th edition as extension of metastatic tumor (tumor present within the confines of the LN and extending through the LN capsule into the surrounding connective tissue, with or without associated stroma reaction) (7). Presence of ENE was examined for each SCC (squamous cell carcinoma)-involved LNs. The exclusion criteria included DM, a previous history of other malignancy within 5 years before study entry except curative treated basal or squamous cell skin cancer or cervical carcinoma *in situ*, a serious concomitant illness that might be aggravated by chemotherapy, pregnancy or breastfeeding, and intestinal obstruction, malabsorption, and any condition that restricts oral medication. Patients fed through nasogastric tubes or gastrostomy tubes without intestinal malabsorption or obstruction were eligible.

CCRT consisted of cisplatin 40 mg/m^2^ weekly or 100 mg/m^2^ every 3 weeks, concomitant with external beam radiotherapy. Radiotherapy was administered with a 6-MV X-ray using intensity-modulated radiotherapy techniques, 2.0 Gy/day per fraction, once daily, five times per week, up to 66 Gy. Patients who received ≥60 Gy were considered as having completed an adjuvant CCRT course.

In the UFTm cohort, participants received UFTm starting from 1 to 2 months after the adjuvant CCRT had ended, with a maintenance duration of 1 year. UFT dose was based on the patient’s body surface area (BSA): 300 mg/day for BSA <1.5 m^2^ and 400 mg/day for BSA ≥1.5 m^2^. Adverse events were evaluated during the monthly follow-ups and were graded using the CTCAE v4.03. If a patient experienced ≥grade 2 adverse events, except anemia, the treatment would be put on hold till the grade of the adverse event was reduced to <2. The UFT dose was then reduced by 100 mg/day for the following treatment. Patients were withdrawn for recurrent disease, intolerable toxicity, or third dose level reduction needed, or if consent was withdrawn at any time during the study.

The conventional follow-up and systemic evaluations in our institute for advanced HNSCC were <3-monthly interval multidisciplinary clinic visits, PET-CT or CT scans (from the head and neck to abdomen) before surgery, 3 months after completion of CCRT, and then every 6 months for 2 years. For the resected pENE+ OSCC in the UFTm cohort, the follow-up consisted of a monthly clinic visit, PET-CT or CT scans (from the head and neck to abdomen) before definitive surgery, within 1 week before adjuvant CCRT, 1–2 months after completing CCRT and before enrolling into the UFTm trial, and then every 3 months during follow-up for 2 years.

OS was the time from the date of definitive surgery until death due to any cause. Event-free survival (EFS) was the time from the date of definitive surgery until the date of relapse or death due to any cause. LRC was calculated from the date of definitive surgery until failure of disease control above the clavicle. DM-free (DMF) survival was defined as the time from the date of definitive surgery until the occurrence of distant metastasis.

Descriptive statistics were applied to patients’ demographic variables, efficacy variables, and adverse events. Inferential statistics were also conducted in this study. The independent two-sample t-test or Wilcoxon rank-sum test was conducted for continuous variables, whereas the chi-square test or Fisher’s exact test was performed for categorical data. Time-to-event data, including OS, EFS, LRC, and DMF, were analyzed using Kaplan–Meier curves and compared with log-rank tests. Univariable and multivariable Cox regression models were applied to assess the relationships between risk factors and treatment outcomes. A two-sided P-value of <0.05 was considered statistically significant. All statistical analyses were performed using the SPSS Statistics for Windows, version 18 (SPSS Inc., Chicago, Ill., USA).

The phase II UFTm trial and this retrospective study were both approved by the institutional review board. Written informed consent was obtained from all patients enrolled in the UFTm trial, whereas the requirement for informed consent from individuals was waived for this retrospective study.

## Results

**Figure 1:**
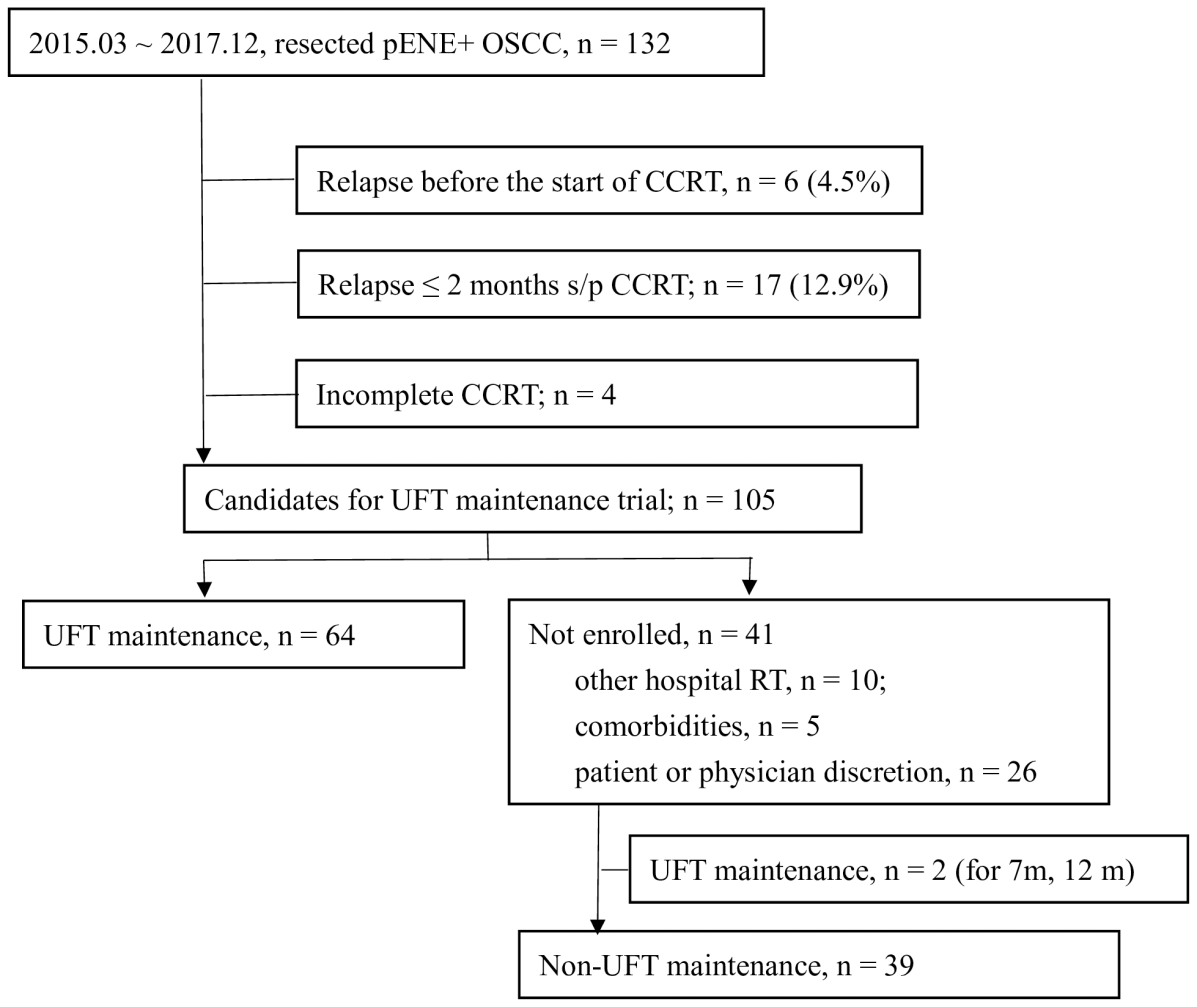
Enrollment of patients. *pENE*, pathologic extranodal extension; OSCC, oral cavity squamous cell carcinoma; *CCRT*, concurrent chemoradiotherapy; *s/p*, status post; *UFT*, tegafur-uracil.

One hundred and thirty-two patients with resected pENE+ OSCC underwent surgery in our institute between March 2015 and December 2017. The data cutoff date was 16 August 2020, and the median follow-up of surviving patients was 43 months (range, 22–65 months). The schema of patient enrollment is shown in **Figure 1**. Six patients (4.5%) relapsed before radiotherapy, and four did not complete their adjuvant CCRT. Furthermore, 17 patients (12.9%) relapsed within 2 months after the end of CCRT. Therefore, 105 patients fulfilled the criteria for the UFTm trial. Of these, 41 did not receive UFTm for the following reasons: 10 patients received adjuvant CCRT at a regional hospital, five patients had comorbidities, and 26 patients decided not to join the trial as per their or the physician’s discretion. There were two patients who received UFTm for 7 or 12 months but did not participate the UFTm trial; both patients had experienced an EFS event at the data cutoff date and were excluded from the cohort of non-UFTm during this analysis. The following analysis describes the 64 patients enrolled for UFTm and 39 non-UFTm patients.

The patients’ characteristics are presented in **Table 1**. The TNM staging was updated in accordance with the American Joint Committee of Cancer 8th edition, which incorporated the depth of invasion and ENE into the T and N staging, respectively (7). There were no statistical differences found for the majority of the characteristics between the two cohorts. However, a trend of longer duration of radiotherapy (54.6 ± 11.8 versus 50.5 ± 8.0 days, p = 0.061) was observed in the non-UFTm cohort. Two patients in the non-UFTm group completed their radiotherapy in 81 and 93 days, and both experienced EFS. After excluding the two patients mentioned above, the RT duration became 50.48 ± 8.05 days in UFTm and 52.81 ± 9.07 days in non-UFTm (p = 0.185). Of the characteristics of LN, the median number of dissected lymph nodes (LN) (55.5 vs. 50.0, p = 0.366), median number of pENE+ LN (2.0 vs. 2.0, p = 0.940), and proportion of subjects with ≧4 pENE+ LN (16 [25%] vs. 9 [23.1%], p = >0.999) were comparable between UFTm and non-UFTm cohorts, respectively. However, more SCC-involved LNs were observed in the non-UFTm cohort (4.0 vs. 2.5, p = 0.038). Forty patients (62.5%) completed the scheduled UFTm course of 1 year. The causes of incomplete course included disease relapse in 14 (21.8%) patients, consent withdrawal in 9 (14.1%) patients, and an adverse event in 1 (1.6%) patient. The adverse events of UFTm are shown in **Table 2**. The most common adverse event was macrocytic anemia. Other adverse events were mostly mild, were of low incidence, and usually could be controlled with dose reduction.

**Table 1:** Patient characteristics.

		UFTm(n = 64)		non-UFTm(n = 39)		P value
Sex, n (%)	Men	58	(90.6)	36	(92.3)	> 0.999
	Women	6	(9.4)	3	(7.7)	
Age (year)	Mean ± SD	49.8	± 8.8	49.9	± 11.5	0.946
ECOG PS, n (%)	1	64	(100)	39	(100)	
CCI score, n (%)	0	44	(68.8)	24	(61.5)	0.164
	1	20	(31.2)	12	(30.8)	
	2	0	(0)	2	(5.1)	
	3	0	(0)	1	(2.6)	
Tumor site, n (%)	Lip	0	(0)	1	(2.6)	0.589
	Tongue	30	(46.9)	20	(51.3)	
	Buccal	20	(31.3)	12	(30.8)	
	Gum	7	(10.9)	2	(5.1)	
	Retromolar	3	(4.7)	3	(7.7)	
	Mouth floor	4	(6.3)	1	(2.6)	
Differentiation, n (%)	Well-moderate	49	(76.6)	30	(76.9)	>0.999
	Poor	15	(23.4)	9	(23.1)	
T, n (%)	1-3	34	(53.1)	15	(38.5)	0.161
	4	30	(46.9)	24	(61.5)	
N, n (%)	2A	11	(17.2)	3	(7.7)	0.240
	3B	53	(82.8)	36	(92.3)	
Stage, n (%)	IVA	11	(17.2)	3	(7.7)	0.240
	IVB	53	(82.8)	36	(92.3)	
No. of dissected LNs	Median, (range)	55.5	(16-185)	50.0	(19-169)	0.366
No. of SCC-involved LNs	Median, (range)	2.5	(1-13)	4.0	(1-15)	0.038
No. of pENE+ LNs	Median, (range)	2.0	(1-12)	2.0	(1-7)	0.940
No. of pENE+ LNs, n (%)	1-3	48	(75)	30	(76.9)	>0.999
	≥4	16	(25)	9	(23.1)	
Margin+, n (%)	Negative	60	(93.8)	35	(89.7)	0.473
	Positive	4	(6.2)	4	(10.3)	
Cisplatin dose (mg/m^2^)*, n (%)	≥200	52	(81.3)	26	(66.7)	0.104
	<200	12	(18.7)	13	(33.3)	
RT dose (Gy), n (%)	60~66	1	(1.6)	2	(5.1)	0.555
	≥66	63	(98.4)	37	(94.9)	
RT duration (days)	Mean ± SD	50.5	± 8.0	54.6	± 11.8	0.061

CCI, Charlson Comorbidity Index; CCRT, concurrent chemoradiotherapy; ECOG, Eastern Cooperative Oncology Group; ECOG PS, ECOG performance status; pENE, pathologic extranodal extension; LN, lymph node N, (from the TNM staging system) number of nearby lymph nodes with cancer; T, (from the TNM staging system) the size and extent of the primary tumor; UFTm, Tegafur-uracil maintenance; RT, radiotherapy. SCC, squamous cell carcinoma.

*Accumulated dose of cisplatin during CCRT.

**Table 2:** Adverse events of UFT maintenance per CTCAE (n = 64).

	Experienced maximal severe grade (%)				
	0	1	2	3	4
**Neutropenia**	67.7	27.4	4.8		
**Anemia**	11.5	75.4	11.5	1.6	
**Thrombocytopenia**	61.3	37.1	1.6		
**Vomiting**	98.4	1.6			
**Mucositis**	48.4	43.8	6.3	1.6	
**Skin**	89.1	10.9			
**Diarrhea**	87.5	12.5			
**Creatinine**	79.0	19.4	1.6		
**Alanine aminotransferase**	82.3	14.5	3.2		

CTCAE, common terminology criteria of adverse event; UFT, tegafur-uracil.

**Figure 2:**
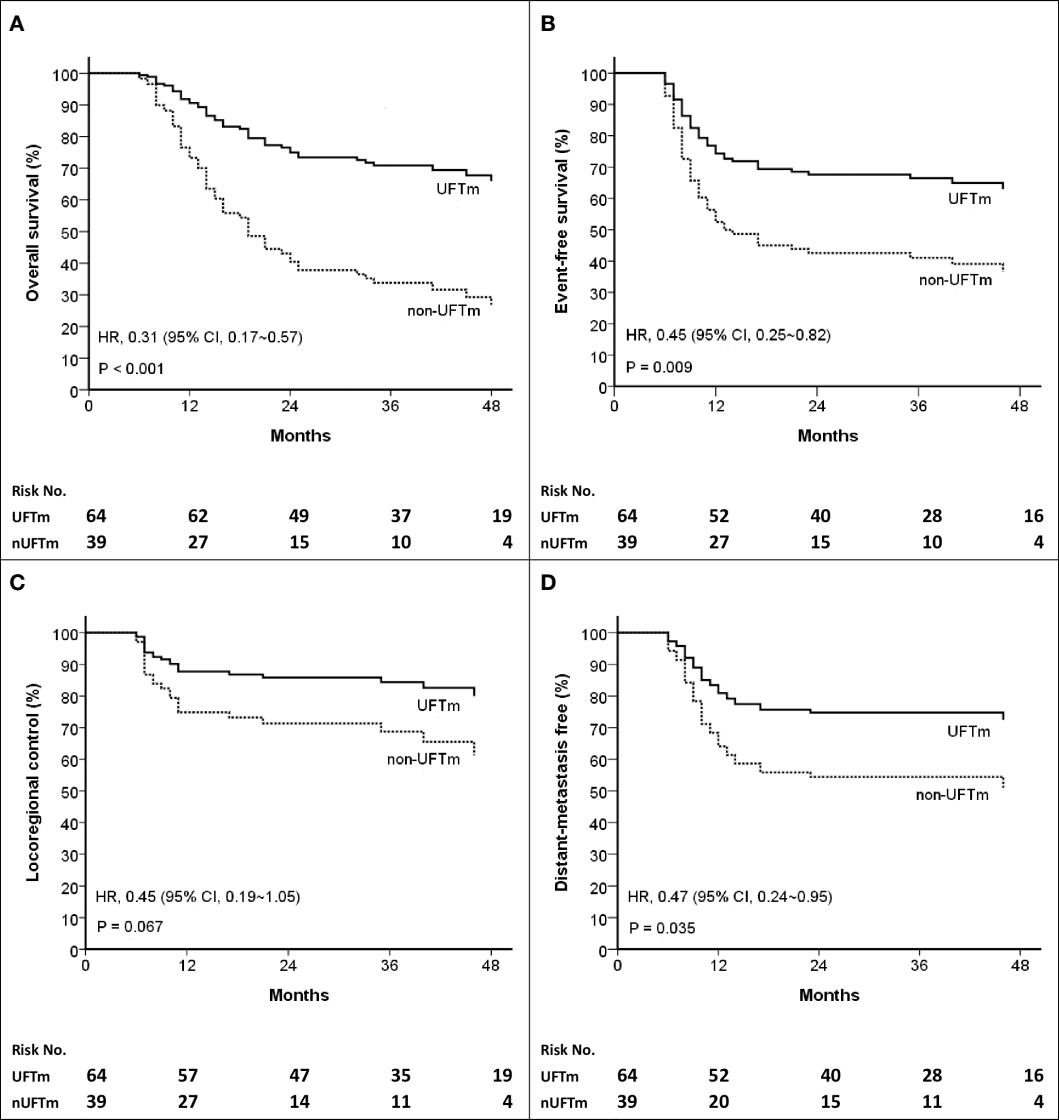
Kaplan–Meier curves of **(A)** Overall survival **(B)** Event-free survival **(C)** Locoregional control, and **(D)** Distant-metastasis free. *HR*, hazard ratio; *CI*, confidence interval; *UFT*, tegafur-uracil; *UFTm*, UFT maintenance; *nUFTm, non-UFTm*.

The events (n, [%]) in UFTm versus non-UFTm till the data cutoff date were death: 21 (32.8%) vs. 25 (64.7%); relapse: 23 (35.9%) vs. 22 (56.4%); locoregional failure: 11 (17.2%) vs. 11 (28.7%); and distant failure: 17 (26.6%) vs. 16 (41.0%). The median duration and hazard ratio (HR) of time-to-event outcomes between UFTm and non-UFTm were OS, undefined vs. 19.0 months (95% CI, 12.9–25.0) (HR 0.31 [95% CI 0.17–0.57], p < 0.001); EFS, undefined vs. 12.0 months (approximately 3.5–20.4) (0.45 [0.25–0.82], 0.009); LRC, both undefined (0.45 [0.19–1.05], 0.067); and DMF, both undefined (0.47 [0.24–0.95], 0.035) (**Figure 2**). The results of univariable analysis (UVA) and multivariable analyses (MVA) are presented in **Table 3**. Due to the limited number of outcome-related events for meaningful analyses in MVA, only potential prognostic factors with statistical significance in UVA were put in the MVA. The unindependent and close relationship between the characteristics of LNs precluded analyzing the impacts of SCC-involved LNs and pENE+ LNs simultaneously in the same MVA with ≧4 pENE+ LNs. The MVA for pENE+ LNs and SCC-involved LNs are presented in **Supplement Tables 1, 2**, respectively. In **Table 3** with the MVA including ≧4 pENE+ LNs as a factor, UFTm remained as an independent factor for OS (0.29 [0.16–0.54], <0.001) and EFS (0.47 [0.26–0.85], 0.012). The Charlson comorbidity index (CCI) scores 1–3 were correlated with distant failure in multivariable analyses (2.04 [1.02–4.08], p = 0.045). The numbers of patients with CCI scores 1–3 were 20 (31.2%) in UFTm and 15 (38.5%) in non-UFTm (p = 0.522) (**Table 1**); of which, the number of patients with distant failure was 10 (50%) in UFTm versus 7 (46.7%) in non-UFTm (p = 0.845). The MVA with cisplatin dose and T4 is presented in **Supplement Table 3**, whereas the MVA with cisplatin dose and stage IVB is shown in **Supplement Table 4**.

**Table 3:** Univariable and multivariable analyses (n = 103).

		OS	EFS	LRC	DM
	n	HR (95% CI),P value	HR (95% CI),P value	HR (95% CI),P value	HR (95% CI),P value
**Univariable**					
UFT maintenance	64	0.32 (0.18-0.57) <0.001*	0.46 (0.25-0.83) 0.009*	0.45 (0.20-1.06) 0.067	0.48 (0.24-0.95) 0.035*
Men	94	0.67 (0.26-1.69) 0.392	0.64 (0.25-1.62) 0.343	0.94 (0.22-4.05) 0.938	0.42 (0.16-1.09) 0.076
Age		1.01 (0.98-1.04) 0.694	1.00 (0.97-1.03) 0.856	0.97 (0.93-1.02) 0.187	1.02 (0.98-1.05) 0.409
CCI = 1-3	35	1.99 (1.11-3.56) 0.021*	2.07 (1.15-3.74) 0.015*	1.58 (0.67-3.73) 0.292	2.41 (1.22-4.78) 0.012*
Tumor site: tongue	50	1.39 (0.78-2.48) 0.272	1.40 (0.78-2.52) 0.259	2.51 (1.02-6.16) 0.045*	1.40 (0.71-2.79) 0.332
Differentiation: poor	24	1.38 (0.71-2.66) 0.339	1.78 (0.95-3.35) 0.073	1.72 (0.70-4.23) 0.236	1.50 (0.70-3.23) 0.300
Stage IVB	89	1.47 (0.58-3.71) 0.419	1.90 (0.68-5.31) 0.220	NA^‡^ 0.988	1.26 (0.44-3.60) 0.660
T4		1.33 (0.74-2.38) 0.345	1.19 (0.66-2.14) 0.566	0.95 (0.41-2.19) 0.901	1.36 (0.68-2.71) 0.385
No. of dissected LNs	103	1.00 (0.99-1.01) 0.955	1.00 (0.99-1.01) 0.623	0.99 (0.98-1.01) 0.281	1.00 (0.99-1.01) 0.695
No. of SCC-involved LNs	103	1.21 (1.10-1.32) <0.001*	1.18 (1.08-1.29) <0.001*	1.20 (1.07-1.35) 0.002*	1.10 (0.98-1.23) 0.096
No. of pENE+ LNs	103	1.19 (1.06-1.35) 0.004*	1.24 (1.09-1.40) 0.001*	1.22 (1.02-1.45) 0.031*	1.14 (0.99-1.30) 0.065
No. of pENE+ ≥ 4	25	2.44 (1.34-4.47) 0.004*	2.73 (1.50-4.98) 0.001*	2.14 (0.89-5.15) 0.090	2.22 (1.09-4.52) 0.029*
Positive margin	8	0.92 (0.29-2.97) 0.892	1.37 (0.49-3.84) 0.545	0.61 (0.08-4.57) 0.634	0.82 (0.20-3.42) 0.784
Cisplatin dose ≧200 mg/m^2†^	78	1.28 (0.63-2.58) 0.493	1.21 (0.60-2.44) 0.601	3.65 (0.85-15.66) 0.081	0.87 (0.40-1.87) 0.716
RT duration		1.00 (0.97-1.03) 0.897	1.01 (0.98-1.04) 0.516	1.02 (0.99-1.06) 0.254	1.00 (0.97-1.04) 0.970
**Multivariable**					
		OS	EFS	LRC	DM
UFT maintenance	64	0.29 (0.16-0.54) <0.0001*	0.47 (0.26-0.85) 0.012*	0.46 (0.19-1.07) 0.070	0.50 (0.25-1.00) 0.051
CCI score = 1-3	35	1.51 (0.83-2.75) 0.177	1.61 (0.88-2.94) 0.123	1.26 (0.53-2.99) 0.596	2.04 (1.02-4.08) 0.045*
Tumor site: tongue	50	1.11 (0.62-2.00) 0.719	1.18 (0.66-2.14) 0.577	2.26 (0.92-5.59) 0.077	1.19 (0.60-2.37) 0.622
No. of pENE+ ≥ 4	25	2.75 (1.46-5.18) 0.002*	2.61 (1.41-4.83) 0.002*	1.97 (0.81-4.83) 0.137	2.13 (1.03-4.39) 0.040*

CCI, Charlson Comorbidity Index; EFS, event-free survival; pENE, pathologic extranodal extension; OS, overall survival; LRC, locoregional control; DM, distant metastasis; DMF, DM free; RT, radiotherapy; NA, not assessable; HR, hazard ratio; UFT, tegafur-uracil. SCC, squamous cell carcinoma.

^†^Accumulated dose of cisplatin during chemoradiotherapy ^‡^All patients with LRC were stage IVb.

*Statistical significance.

**Figure 3:**
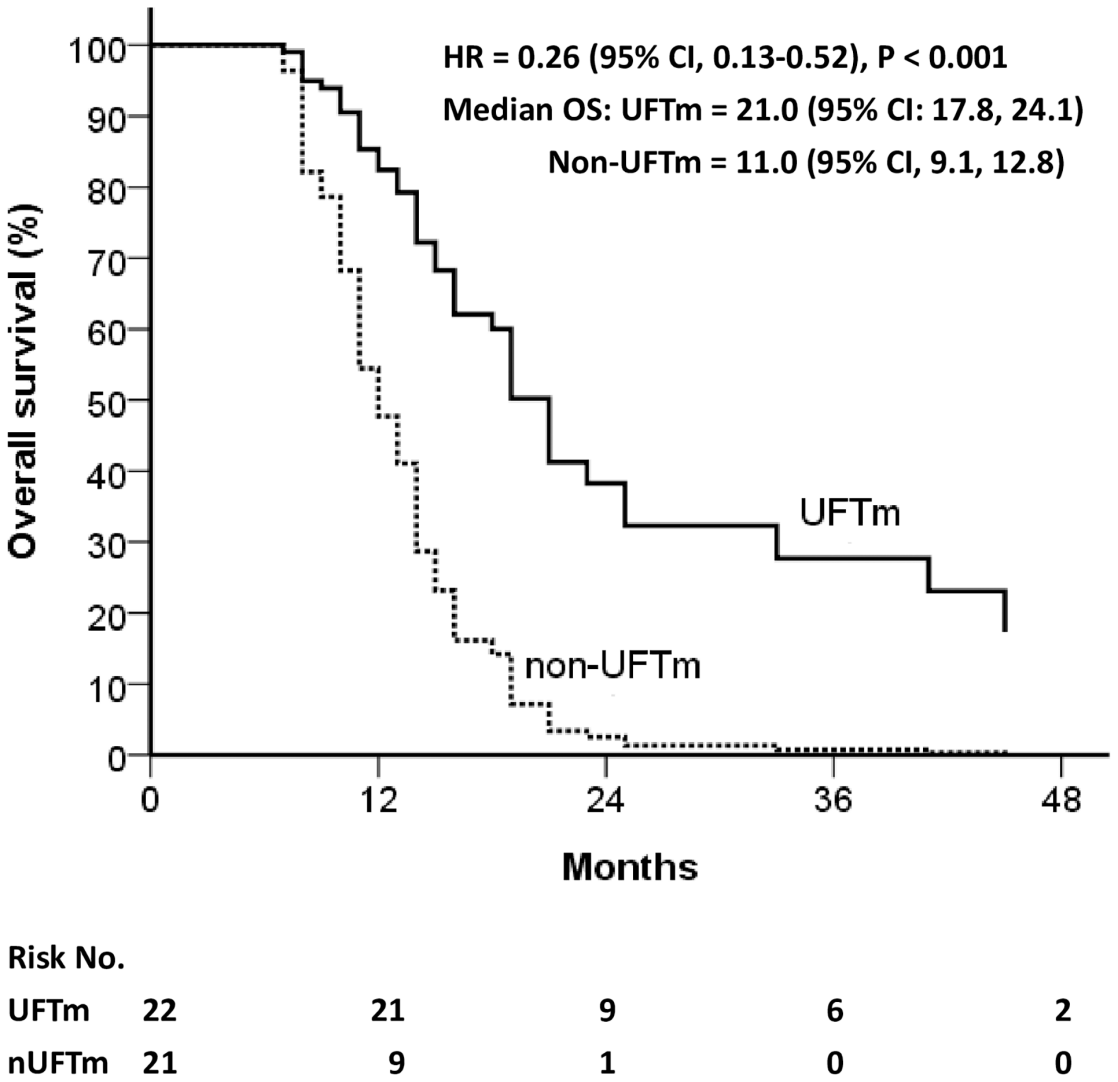
Overall survival of relapsed patients. *CI*, confidence interval; *HR*, hazard ratio; *OS*, overall survival; *UFT*, tegafur-uracil; *UFTm*, UFT maintenance.

The disease course of the resected pENE+ OSCC was followed up from the date of definitive surgery (n = 132). Relapse occurred continuously before CCRT (n = 6, 4.5%), shortly after CCRT ended (n = 17, 12.9%), and during the follow-up with or without UFTm. The locoregional vs. distant failure rate in relapse before CCRT was 100% vs. 50%, while those in relapse after CCRT was 61% vs. 83%. The relapse rate for patients included in the present study was 35.9% in the UFTm group and 56.4% in the non-UFTm group (p = 0.042). In these relapsed patients, there was no significant difference with reference to locoregional failure (47.8% vs. 50.0%, p = 0.884) and DM (73.9% vs. 72.7%, p = 0.928) between the UFTm vs. non-UFTm groups. The median OS for relapsed patients was 21.0 (95% CI, 17.8–24.1) months in the UFTm vs. 11.0 (9.1–12.8) months in the non-UFTm group (HR = 0.26 [0.13–0.52], p < 0.001) as shown in **Figure 3**. For patients with DM, metastasectomy for oligometastasis was done in nine (53%) patients in the UFTm group and one (6%) patient in the non-UFTm group. In the UFTm group, there was no difference in OS in patients with distant failure that did or did not undergo the metastasectomy (p = 0.783).

## Discussion

The phase II UFTm trial was designed to reduce the 2-year distant failure rate from 26% to 13% in patients with resected pENE+ OSCC status post adjuvant CCRT and UFTm. This estimation was referred to from the results of adjuvant CCRT with docetaxel plus cetuximab in the RTOG 0234 trial (11). However, the 2-year distant failure rate of 25.8% in the UFTm group, although much lower than the 44.2% in the non-UFTm group, implies that the endpoint of the phase II trial was not met. The incidence of distant failure was underestimated in this study. This discrepancy may be because patients with pENE+ were 59% in RTOG 0234 but 100% in the current UFTm trial.

In this study, patients in the phase II UFTm trial had significantly better OS, EFS, and DMF and a trend of better LRC than those of the non-UFTm patients. These results were different from the randomized trial of the Head and Neck UFT study group that used UFTm 300 mg/day for 1 year in 398 patients, which showed no difference in OS and relapse-free survival but showed a significant reduction only in DM as the first relapse from 14.6% to 7.9% (p = 0.03) (12). The efficacy of UFTm on other time-to-event outcomes beyond the distant failure was also shown in retrospective studies for high-risk advanced HNSCC (18, 19). The first possible reason for the difference may be that the Head and Neck UFT trial study group enrolled patients with less advanced HNSCC (about 70% stages II and III) and received no adjuvant treatment except with/without UFTm. Their major outcome determinant might be the first relapse of locoregional disease (19.5%) rather than DM (9.8%). Our trial, with a primary focus on the effect of UFTm on distant failure, enrolled 100% of stage IV patients with distant recurrence as the major first-relapse site (32%) rather than locoregional (21.4%). This enriched design may more clearly reveal the effect of UFTm on distant failure and overall outcomes. A possible second reason is the dosage and duration of UFTm. In addition to our UFTm of 400 mg/day for 1 year, a higher dose and longer duration of UFTm after tumor resection had demonstrated prolonged survival in patients with stage III rectal cancer (400 mg/m^2^/day for 1 year) (20), T2N1–2 gastric cancer (360 mg/m^2^/day for 16 months) (21), and stage I lung cancer (250 mg/m^2^/day for 2 years) (22). However, in patients with distant failure in the current trial, 88% (15/17) in the UFTm group and 100% (16/16) in the non-UFTm group developed distant failure within the scheduled 1-year UFTm period. Therefore, we recommend a prescription of 1-year of UFTm.

Another interesting finding was that, besides the reduced relapse rate, the median OS for relapsed patients was 21.0 (95% CI, 17.8–24.1) months in the UFTm group vs. 11.0 (95% CI, 9.1–12.8) months in the non-UFTm group, p < 0.001 (**Figure 3**). Although the UFTm cohort had less relapse, the rate of locoregional and distant failure in the relapsed patients was similar between the UFTm and non-UFTm cohorts. However, in patients with DM, metastasectomy for oligometastasis was feasible in nine (53%) patients in the UFTm group and one (6%) patient in the non-UFTm group. This implies that UFTm may not just reduce relapse but also modify the disease course in relapsed patients. UFTm can be viewed as a type of metronomic chemotherapy, and it provides its antitumor response through its anti-angiogenesis effect (23, 24), direct anticancer activity (14), and possible immuno-stimulatory effects (25, 26). These interactions between the tumor cells and their microenvironment may render high-risk patients with less distant failure, fewer polymetastases, and more oligometastasis that can be salvaged by local therapy (27, 28).

Although our study suggested that UFTm improved the outcomes of resected pENE + OSCC, there were a considerable number of patients who relapsed before the scheduled UFTm. Six patients (4.5%) relapsed before radiotherapy, and 17 (12.9%) patients relapsed within 2 months after CCRT. Seventy-five percent of these patients with early relapse had DM. A previous study used an experimental breast-cancer model system that linked postsurgery systemic inflammatory response to tumor cell outgrowth at distant anatomical sites, and these tumor outgrowths were otherwise restricted by a tumor-specific T-cell response and perioperative anti-inflammatory treatment (29). The potential for preventing disease progression and improving DFS by the perioperative oral metronomic chemotherapy with methotrexate and celecoxib has been suggested in a small matched-pair analysis (30). Whether perioperative anti-inflammatory treatment plus UFT can extend the potential benefit of UFTm to the early relapse patient is worth further investigation.

This study had several limitations. First, there was an underestimation of the distant failure rate for resected pENE+ OSCC. Second, matched controls were not used; instead, controls were substituted by conducting comparisons using a patient cohort of non-UFTm during the enrollment period of the UFTm trial. Although the distant failure rate is an objective disease-specific endpoint, the indirect comparisons may harbor the potential of selection bias from an unclarified patient and tumor characteristics. Although patients with CCI scores 1–3 and their distant failure were distributed equally between the two cohorts, the independent role of CCI scores 1–3 with distant failure should still be noticed in future investigation. The longer duration of radiotherapy in non-UFTm may raise a concern about longer RT duration with worse outcomes. However, if the two patients with 81 and 93 days of RT duration were excluded, the RT duration became (means [ ± SD]) 50.48 ± 8.05 days in UFTm and 52.81 ± 9.07 days in non-UFTm (p = 0.185). Both patients were in EFS, and excluding them from the analysis may bias and magnify the better outcome of UFTm. RT duration has also been shown to not be correlated with outcomes in UVA. In our previous study, the recursive partitioning analysis for 201 resected pENE+ OSCC patients revealed that no adjuvant CCRT and ≧4 pENE+ LNs were the most important factors correlated with worse disease-specific survival. The treatment selection bias in the current study was minimized because only patients who had completed their adjuvant CCRT were included, and the proportion of patients with≧4 pENE+ LNs was also similar between the two cohorts in the current study. Also, compared with patient and tumor characteristics within MVA in **Table 3**, UFTm claimed its independent role on OS and EFS.

## Conclusions

UFTm is a safe and potentially effective treatment for improving oncologic outcomes of resected pENE+ OSCC. The high incidence of early relapse and more distant failure with the oligometastatic disease in UFTm during the current trial warrant other potential interventions to improve the outcomes of patients with resected pENE+ OSCC.

## Data availability statement

The raw data supporting the conclusions of this article will be made available by the authors, without undue reservation.

## Ethics statement

The studies involving human participants were reviewed and approved by Chang Gung Medical Foundation Institutional Review Board https://www1.cgmh.org.tw/intr/intr1/c0040/. The patients/participants provided their written informed consent to participate in this study.

## Author contributions

H-MW conceived the study and participated in the design and administrative coordination. P-WH, C-YL, C-HH, C-LH, S-FH, and C-TLiao participated in the provision of study materials or patients. L-YL is responsible for pathologic diagnosis and evaluation for pENE. All authors participated in the collection and assembly of data, data analysis, and preparation of the manuscript. All authors read and approved the final manuscript.

## Conflict of interest

The authors declare that the research was conducted in the absence of any commercial or financial relationships that could be construed as a potential conflict of interest.

## Publisher’s note

All claims expressed in this article are solely those of the authors and do not necessarily represent those of their affiliated organizations, or those of the publisher, the editors and the reviewers. Any product that may be evaluated in this article, or claim that may be made by its manufacturer, is not guaranteed or endorsed by the publisher.
