# Supplementary material for: Maintenance tegafur-plus-uracil after adjuvant concurrent chemoradiotherapy may improve outcome for resected oral cavity squamous cell carcinoma with extranodal extension
Source: Front Oncol. 2022 Sep 28;12:866890. doi: 10.3389/fonc.2022.866890 (PMC9554642; doi:10.3389/fonc.2022.866890)
Supplement: Supplementary file 1 [file DataSheet_1.pdf]

Supplement Table 1. Multivariable analysis to include the number of pENE+ LNs as a factor

Multivariable

		OS	EFS	LRC	DM
	n	HR (95% CI), P value	HR (95% CI), P value	HR (95% CI), P value	HR (95% CI), P value
UFT maintenance	64	0.30 (0.16-0.55) <0.0001*	0.47 (0.26-0.86) 0.015*	0.46 (0.19-1.07) 0.007	0.50 (0.25-1.01) 0.054
CCI score = 1-3	35	1.62 (0.89-2.93) 0.113	1.69 (0.93-3.09) 0.087	1.28 (0.54-3.02) 0.576	2.14 (1.07-4.29) 0.032”
Tumor site: Tongue	50	1.05 (0.58-1.90) 0.886	1.13 (0.62-2.05) 0.693	2.18 (0.88-5.40) 0.094	1.14 (0.56-2.29) 0.724
No. of pENE+ LNs	103	1.24 (1.09-1.42) 0.002*	1.24 (1.08-1.42) 0.002*	1.20 (0.99-1.44) 0.060	1.15 (0.99-1.34) 0.066

Abbreviations: ***CCI***, Charlson Comorbidity Index; ***pENE***, pathologic extranodal extension; ***UFTm***, Tegafur-uracil maintenance

*Statistical significance

Supplement Table 2. Multivariable analysis to include the number of SCC-involved LNs as a factor

**Multivariable**					
		OS	EFS	LRC	DM
	n	HR (95% CI), P value	HR (95% CI), P value	HR (95% CI), P value	HR (95% CI), P value
UFT maintenance	64	0.37 (0.20-0.68) 0.001*	0.59 (0.32-1.10) 0.098	0.57 (0.24-1.37) 0.207	0.57 (0.28-1.16) 0.122
CCI score = 1-3	35	1.52 (0.83-2.77) 0.177	1.66 (0.90-3.04) 0.103	1.27 (0.54-3.02) 0.584	2.09 (1.04-4.20) 0.039*
Tumor site: Tongue	50	1.08 (0.60-1.94) 0.803	1.20 (0.67-2.16) 0.547	2.17 (0.88-5.36) 0.092	1.22 (0.61-2.44) 0.567
No. of SCC-involved LNs	103	1.19 (1.07-1.31) 0.001*	1.15 (1.04-1.26) 0.005*	1.17 (1.03-1.32) 0.015	1.06 (0.94-1.20) 0.333

Abbreviations: ***CCI***, Charlson Comorbidity Index; ***SCC***, squamous cell carcinoma; ***UFTm***, Tegafur-uracil maintenance

*Statistical significance

Supplement Table 3. Multivariable analysis to include cisplatin dose and T4 as factors

**Multivariable**					
		OS	EFS	LRC	DM
	n	HR (95% CI),<br>P value	HR (95% CI),<br>P value	HR (95% CI),<br>P value	HR (95% CI),<br>P value
UFT maintenance	64	0.27 (0.14-0.51)<br>< 0.0001*	0.43 (0.23-0.81)<br>0.008*	0.35 (0.14-0.84)<br>0.020*	0.52 (0.25-1.07)<br>0.077
CCI score = 1-3	35	1.46 (0.79-2.70)<br>0.224	1.53 (0.82-2.86)<br>0.178	1.14 (0.46-2.80)<br>0.781	2.09 (1.03-4.26)<br>0.042*
Tumor site: Tongue	50	1.15 (0.64-2.07)<br>0.640	1.22 (0.68-2.21)<br>0.509	2.42 (0.98-5.99)<br>0.056	1.17 (0.58-2.35)<br>0.658
No. of pENE+ LN ≥ 4	25	2.76 (1.41-5.41)<br>0.003*	2.75 (1.41-5.35)<br>0.003*	2.28 (0.86-6.03)<br>0.097	2.04 (0.94-4.43)<br>0.072
Cisplatin dose ≥ 200 mg/m^2	78	1.60 (0.78-3.27)<br>0.201	1.34 (0.65-2.74)<br>0.431	4.57 (1.05-19.94)<br>0.044*	0.92 (0.42-2.00)<br>0.830
T4	54	0.95 (0.50-1.80)<br>0.869	0.83 (0.43-1.61)<br>0.576	0.58 (0.22-1.55)<br>0.276	1.14 (0.53-2.45)<br>0.740

Abbreviations: ***CCI***, Charlson Comorbidity Index; ***pENE***, pathologic extranodal extension; ***UFTm***, Tegafur-uracil maintenance

*Statistical significance

Supplement Table 4. Multivariable analysis to include cisplatin dose and Stage IVB as factors

Multivariable

		OS	EFS	LRC	DM
	n	HR (95% CI), P value	HR (95% CI), P value	HR (95% CI), P value	HR (95% CI), P value
UFT maintenance	64	0.27 (0.14-0.50) < 0.0001*	0.45 (0.25-0.84) 0.011*	0.39 (0.17-0.93) 0.033*	049 (0.24-1.00) 0.050*
CCI score = 1-3	35	1.52 (0.83-2.78) 0.179	1.59 (0.87-2.92) 0.136	1.32 (0.56-3.11) 0.532	2.12 (1.04-4.30) 0.039*
Tumor site: Tongue	50	1.13 (0.63-2.04) 0.676	1.21 (0.67-2.19) 0.531	2.34 (0.95-5.76) 0.064	1.16 (0.58-2.32) 0684
No. of pENE+ LN ≥ 4	25	2.81 (1.47-5.41) 0.002*	2.51 (1.33-4.73) 0.005*	1.86 (0.76-4.54) 0.172	2.27 (1.07-4.82) 0.034*
Cisplatin dose ≥ 200 mg/m^2	78	1.61 (0.78-3.31) 0.195	1.32 (0.64-2.70) 0.450	4.32 (1.00-18.73) 0.051	0.92 (0.42-2.01) 0.829
Stage IVB	89	0.78 (0.29-2.11) 0.619	1.13 (0.38-3.37) 0.821	-[#]	0.71 (0.23-2.20) 0.553

Abbreviations: ***CCI***, Charlson Comorbidity Index; ***pENE***, pathologic extranodal extension; ***UFTm***, Tegafur-uracil maintenance

*Statistical significance

[#]For LRC analysis, stage IVB was not included for multivariable analysis because all patients who had an LRC event were of stage IVB.
